# Guideline for improving the reliability of Google Ngram studies: Evidence from religious terms

**DOI:** 10.1371/journal.pone.0213554

**Published:** 2019-03-22

**Authors:** Nadja Younes, Ulf-Dietrich Reips

**Affiliations:** Department of Psychology, University of Konstanz, Konstanz, Germany; University of Vermont, UNITED STATES

## Abstract

The Google Books Ngram Viewer (Google Ngram) is a search engine that charts word frequencies from a large corpus of books and thereby allows for the examination of cultural change as it is reflected in books. While the tool’s massive corpus of data (about 8 million books or 6% of all books ever published) has been used in various scientific studies, concerns about the accuracy of results have simultaneously emerged. This paper reviews the literature and serves as a guideline for improving Google Ngram studies by suggesting five methodological procedures suited to increase the reliability of results. In particular, we recommend the use of (I) different language corpora, (II) cross-checks on different corpora from the same language, (III) word inflections, (IV) synonyms, and (V) a standardization procedure that accounts for both the influx of data and unequal weights of word frequencies. Further, we outline how to combine these procedures and address the risk of potential biases arising from censorship and propaganda. As an example of the proposed procedures, we examine the cross-cultural expression of religion via religious terms for the years 1900 to 2000. Special emphasis is placed on the situation during World War II. In line with the strand of literature that emphasizes the decline of collectivistic values, our results suggest an overall decrease of religion’s importance. However, religion re-gains importance during times of crisis such as World War II. By comparing the results obtained through the different methods, we illustrate that applying and particularly combining our suggested procedures increase the reliability of results and prevents authors from deriving wrong assumptions.

## Introduction

Since its launch in 2010, the possibilities and limitations of using the Google Books Ngram Viewer (Google Ngram) for research purposes have been controversially discussed. Although the large number of Google Ngram studies indicates scientific recognition, several papers rightly address methodological issues (see, e.g., [1,2,3]). Yet, no set of applicable solutions is given. Our present article aims at mitigating concerns about the trustworthiness of Google Ngram studies by presenting a guideline on different hands-on procedures that can increase reliability.

Google Ngram is a search engine that charts word frequencies from a large corpus of books that were printed between 1500 and 2008. The tool generates charts by dividing the number of a word’s yearly appearances by the total number of words in the corpus in that year. Thereby a book’s content is split into case-sensitive text blocks–so called *n-grams*. The word “book”, for example, is a 1-gram, whereas “holy book” is a 2-gram and so on, up to a maximum of five words. Because early decades contain significantly fewer books, the overall corpus of Google Ngram becomes sufficiently large for scientific use by the year 1800 [4]. When the tool was released in 2010, the total corpus consisted of more than 5 million books, covering the languages English, French, Spanish, German, Chinese, Russian, and Hebrew. These books were drawn from over 40 different university libraries [4]. Since the corpus’ latest update in 2012, users can access 22 different sub-corpora, encompassing 8 million books in total. The new version is characterized by improved optical character recognition (OCR) as well as better underlying library and publisher metadata [5]. By now, two corpora for each of the above-mentioned languages exist–an old version and an updated version. One additional corpus contains Italian books. Four further corpora split English into British and American English. Finally, there are two fiction corpora, which include predominately English fiction books and one corpus, called “English one Million”, which includes a balanced text-collection of 6000 English language books, published between 1500 and 2008, and chosen from any one year.

When Google Ngram was released, Michel et al. [4] argued that one of the tool’s main opportunities for scientific purposes is the hands-on quantification of cultural development that can be measured through changes in word frequencies. Related to the tool’s free and easy access, Google Ngram has further contributed to an ongoing debate–the ease of replicability. By now, several dozen studies have embraced Google Ngram as an opportunity to gain insight into the development of cultural changes (see Table A in S1 Appendix for an overview of psychological Google Ngram research, published between 2010 and 2018). A vast strand of research hereby empirically tests theoretical predictions on cultural changes with a particular focus on individualism and collectivism. Twenge et al. [6,7], for example, report an increase in the frequency of individualistic words and phrases in American books. Kesebir and Kesebir [8] document a decline in the frequency of moral terms, while Twenge et al. [9] highlight an increase in the frequency of swear words. In addition, Greenfield’s [10] findings suggest a relationship between increased individualism and ecological changes in the US and UK, as derived from word frequency changes in the American and British English corpora. The usage of Google Ngram has, however, not been limited to American and British English books. By tracing concepts of folk believes or personal pronouns, several studies document rising individualism also for rather collectivistic societies like China [11,12,13]. Further, such developments were also examined for German-speaking countries [14] and (Soviet) Russia [15,16]. Besides these types of studies, Google Ngram research shed light on topics such as gender differences [17,18,19], the expression of emotions [20,21,22], personality [23,24], and cognition [25,26,27].

In spite of Google Ngram’s rising acceptance among researchers, critics justifiably do not grow tired in highlighting potential problems that may challenge the reliability of existing results. Main points of criticism relate to insufficient OCR, particularly with respect to semantic scanning errors (which can affect words such as *fail* and *sail* due to similarities in the letters “f” and “s”), and messy metadata that may lead to the display of word frequencies in wrong or unrelated time intervals. Another often heard critique refers to the overall corpora’s large proportion of scientific literature and the possibility for single authors to heavily influence the data set with specific words and phrases (see, e.g., [1,2,3]).

In this guideline, we propose how to address these concerns by introducing several methodological procedures such as cross-validations via the examination of different language corpora, the use of word inflections and synonyms, as well as the use of a newly-developed standardization procedure that all aim at increasing the reliability of Google Ngram studies. Further, in a step-by-step example we apply all of these procedures by investigating cross-cultural religious trends over the last century (1900–2000) for cultures whose language is covered by Google Ngram and based on a Latin alphabet. To be able to derive assumptions from language- to country-level, we focus primarily on American and British English, German, and Italian, but consider multinational languages like Spanish and French to verify the general validity of our findings.

## Assessing the development of religion via Google Ngram

While the world population almost quintupled from approximately 1.5 billion in 1900 to 6.9 billion in 2010, the number of people reporting to have no religion increased by more than 265 times (from 3 million in 1900 to 797 million in 2010) over the same period [28]. In other words, nowadays, roughly one in every ten persons on the globe reports to have no religion compared to one in every 500, a hundred years ago. This development stands in line with the vast amount of literature that emphasizes the decline of collectivistic values such as religious belonging, in the wake of an increasing industrialization (e.g., [10,29]; see also [30] for a review on collectivism and individualism). Inglehart and Baker’s [31] profound study on *cultural change and the persistence of traditional values* confirms these shifts, but also points out that cultural changes can be bidirectional. Using Google Ngram, Younes and Reips [14] report on such a bidirectional change by showing an overall decline for collectivistic German words but a reversal during the time of the Nazi Regime and World War II (WWII), indicating the Germans’ movement towards a more collectivistic society during that time. Accordingly, Pargament et al. [32] suggest that especially in times of crises such as wars, people cling to religious beliefs. Along these lines, several studies also mention the importance of religion as a coping mechanism during traumatic and stressful life events (e.g., [33]; see also [34] for a review).

We believe that the cross-cultural development of religion constitutes a suitable research topic to present how Google Ngram can be used for scientific purposes and what can be done to improve the investigation method of previous Google Ngram studies. Because we expect a theory-based cross-cultural decline in the importance of religion, we hypothesize that the relative frequency of religious terms in books covered by Google Ngram will decrease over time. We further hypothesize that–despite an overall decrease in the frequency of religious terms–there will be a reversal during crises that affect a large proportion of a country’s population. In particular, based on the German population’s complete involvement in the severe and lengthy crisis at the time of the Nazi Regime, i.e., in the years before and during WWII, we expect to observe a positive trend for religious German terms during the years 1933 and 1945. Throughout the study, we refer to this time span as WWII.

## Method and results

This section describes how to conduct a Google Ngram study. We discuss several methodological improvements exemplarily in a cross-cultural setting by analyzing the development of frequencies for 20 religious terms in the American and British English, German, and Italian Google Ngram corpora. We use the latest version of each corpus and restrict our investigation period to the last century because the number of books before 1900 is relatively small and recently published books, i.e., those after the year 2000, may still need to be included in the data set [20]. For reasons of comparability to previous studies, we focus on 1-grams.

### Collection of words and verification procedure

We obtain a set of 23 religious English terms from Ritter and Preston [35] who surveyed the literature of words that were used to prime religious concepts. Their list contains common nouns that represent general religious concepts. To facilitate the collection of religious German and Italian words and to establish a word identification process that follows clear criteria, we translated the religious English terms into German and Italian using PONS online dictionary (https://en.pons.com/translate). We excluded the word “faith” because the concept is already represented by the word “belief”. Further, we excluded the words “holy day” and “scripture” because the first expression consists of two words and the translation of the latter leads to terminologies consisting of two words (e.g., “Heilige Schrift” in German and “Sacre Scritture” in Italian). As suggested by Zeng and Greenfield [13] and Younes and Reips [14], we recommend asking several native speakers to check independently from each other whether the respective translations are in line with the original terms. In our setting, we asked two native speakers per language, who are also proficient in English. All agreed that the presented terms were translated in an adequate manner. Table 1 shows the final list of words and their corresponding translations.

**Table 1:** List of religious English terms with their German and Italian translations.

Original	German	Italian
altar	Altar	altare
angel	Engel	angelo
belief	Glaube	fede
clergy	Geistlichkeit	clero
creed	Überzeugung	credo
doctrine	Doktrin	dottrina
God	Gott	Dio
heaven	Himmel	paradiso
miracle	Wunder	miracolo
pilgrimage	Pilgerfahrt	pellegrinaggio
prayer	Gebet	preghiera
prophet	Prophet	profeta
religion	Religion	religione
revelation	Offenbarung	rivelazione
ritual	Ritual	rituale
saint	Heiliger	santo
sermon	Predigt	predica
shrine	Schrein	santuario
soul	Seele	anima
spirit	Geist	spirito

### Baseline analysis

After extracting word frequencies from Google Ngram, prior studies have investigated cultural changes by examining the correlation coefficients between word frequencies and years. Based on the idea that the natural frequency of a word is relevant for assessing cultural change, it is reasonable to sum up the frequencies of single words per year (and language), and to run an aggregated correlational analysis (see, e.g., [6,7,9,18]). In this respect, the more frequent a word, the larger its proportional influence. By analyzing the summed frequencies of our religious words (by year and language), we observe a negative correlation between the years and the religious terms’ frequencies for American English (r = -0.89, p<0.001), British English (r = -0.96, p<0.001), German (r = -0.47, p<0.001), and Italian (r = -0.64, p<0.001). In contrast, investigating the development of religious terms in times of crisis such as WWII, German terms show a significant positive trend (r = 0.79, p<0.01). The findings suggest that religion became less important over time but showed more prevalence during a time of existential crisis (see Figures A-H in S1 Appendix for visual inspection).

### Procedure I: Multiple languages

As highlighted by our current analysis, one big advantage of Google Ngram is the possibility to compare cultural changes in a cross-cultural setting. Although transferring assumptions from language- to country-level, particularly for multinational languages, can be a difficult procedure, several studies suggest comparing differences and similarities of several languages to derive assumptions on the general validity of a certain theory or concept [36,37,38]. Following this reasoning, we further examined religious words’ frequencies during the years 1900 to 2000 using the Spanish and French corpora. Hence, we first translated the original religious English terms into Spanish and French using PONS. Again, two French- and two Spanish-speaking natives confirmed our translations. Table B in S1 Appendix displays the original English terms with their respective translations to Spanish and French. In line with our initial findings, religious words in Spanish (r = -0.47, p<0.001) and French (r = -0.83, p<0.001) show a significant negative trend over time (see Figures I-L in S1 Appendix for visual inspection).

### Procedure II: English fiction corpus

So far, the majority of Google Ngram studies focuses on the English language, i.e., by investigating the American and British English corpora. Besides the traditional English corpora, Google Ngram also offers access to an English fiction corpus. The corpus is limited to fiction books in the Google database and does not distinguish between American and British English. Although it might seem counterintuitive, the fiction corpus may provide an additional opportunity to test the reliability of derived assumptions on cultural changes from the American and British English corpora. Following the argument that “a fiction writer may aim to capture realistic modern dialogue” ([7], p. 407), cultural changes should be also reflected in the fiction corpus—at least to some extent. Because the traditional corpora contain a large amount of scientific text, particularly throughout the 1900s [2], Virues-Ortega and Pear [27] argue that fiction books are less influenced by scientific trends and may therefore provide more general results. Accordingly, several studies used the fiction corpus to test the robustness of their findings. For instance, Twenge et al. [7] confirm their findings for a decrease in first person plural pronouns and an increase in first person singular pronouns by using the fiction corpus. Acerbi et al. [20] document an overall decrease in the use of mood words, which is also reflected in the fiction corpus. By analyzing manifestations of individualism, Twenge et al. [9] confirm the significant increase in the use of swear words also for the fiction corpus. Dependent on the particular analysis, contemplating fictional literature may provide additional insights. Brysbaert et al. [39], for example, report that word frequencies extracted from the fiction corpus predict word processing better than word frequencies obtained from the regular corpora. Because the fiction corpus is only available in English, we investigated the trend pattern for the original religious English terms. By using the fiction corpus, the significant negative overall trend (r = -0.96, p<0.001) can be confirmed (see Figures M and N in S1 Appendix for visual inspection).

### Procedure III: Word inflections

**Fig 1:**
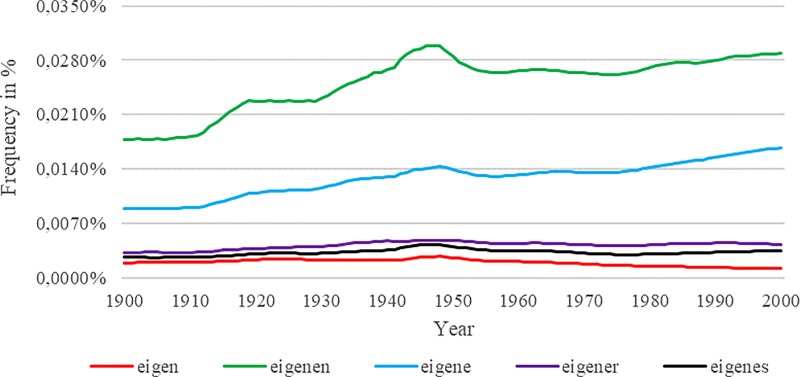
Higher frequency inflections for the German word “eigen”.

Using Google Ngram to analyze long-term relationships between ecological and cultural changes in German-speaking countries, Younes and Reips [14] report that in spite of the theory-based prediction of an increase, the relative frequency of the individualistic word “eigen” (personal/individual) dropped over time. As highlighted by Fig 1, all of the word’s higher frequency inflections (i.e., “eigenen”, “eigene”, “eigener”, or “eigenes”) display, however, the expected rise over the course of time.

Because “the most robust historical trends are associated with frequent n-grams” ([4], Supplementary Online Material, p.12), one potential reason for the counter-predictive trend displayed for the word “eigen” may be the word’s comparatively low overall frequency. Considering the use of the tag “_INF” may help to address that issue in a systematic way. Applying the tag, Google Ngram provides graphs for yet available inflections of a certain word. To obtain inflections, Google Ngram uses Wiktionary entries (www.wiktionary.org) and supplement them with automatically generated inflection tables. “Because Wiktionary is an evolving resource, results for a particular [Google Ngram] query may change over time” ([40], p. 4). In this respect, the tool allows for a direct comparison of inflection frequencies. Thus, the feature is not only helpful for identifying words of highest frequency within a similar group, but also highlights inconsistencies of lower frequency words such as in the case of “eigen”.

**Fig 2:**
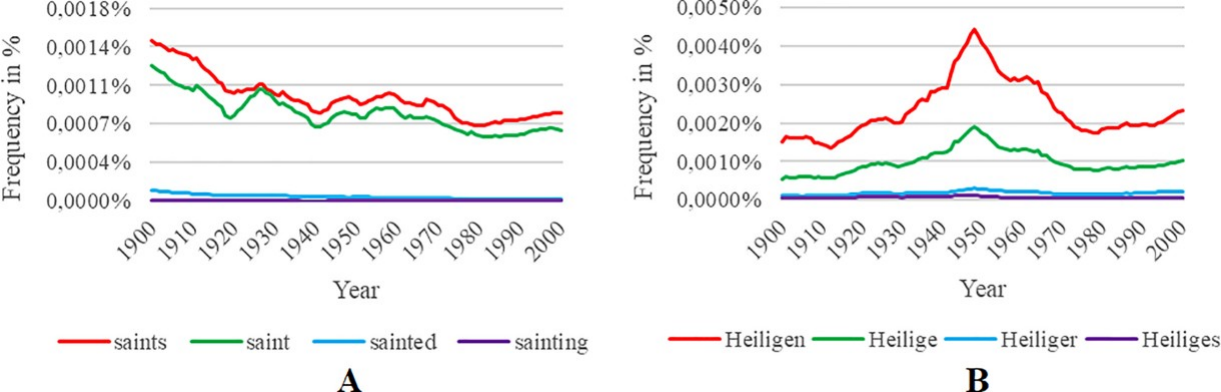
Frequencies of inflections for the word “saint”. Frequencies of given inflections for the word “saint” using the American English corpus (A) and the three most frequent inflections for the German translation “Heiliger” (B).

In this analysis, we checked the consistency between and the frequencies of the original and translated terms and their respective inflections. We exemplify the procedure on the word “saint”. Searching for the word’s inflections (by using “saint_INF”) in the American and British English corpora yields three modifications (“saints”, “sainted”, and “sainting”). As indicated by Fig 2(A), for the American English corpus, the term “saints” shows a similar development as “saint” but exhibits a higher relative frequency. The picture for British English is similar. However, the terms “saint” and “saints” have an almost identical frequency. In contrast, searching for inflections of the respective German translation “Heiliger” yields 13 inflections. Fig 2(B) displays the three most frequent inflections (“Heiligen”, “Heilige”, and “Heiliges”) and thereby illustrates that the term “Heiliger” shows a similar pattern but an approximately three times lower relative frequency than “Heilige” and a 13 times lower relative frequency compared to the most frequent word “Heiligen”.

Finally, no higher frequency inflections are given for the Italian word “santo”. While all of our originally selected terms display similar patterns as their semantically similar inflections, the original English terms as well as our translations were not always those with the highest frequency compared to their inflections. Table 2 provides an overview of those terms for which we found higher frequency inflections as well as the ratios between the frequencies of higher frequency inflections and the frequencies of their original counterparts.

**Table 2:** Overview of original words and their higher frequency inflections.

American English			British English		
Original	High	Ratio	Original	High	Ratio
angel	angels	1.08	angel	angels	1.12
saint	saints	1.18			
German			Italian		
Original	High	Ratio	Original	High	Ratio
Engel	Engels	2.28	angelo	angeli	1.52
Glaube	Glauben	1.91			
Prophet	Propheten	2.54			
Heiliger	Heiligen	13.10			

The columns “Original” present original words, whereas the columns “High” present the original words’ higher frequency inflections. The columns “Ratio” display the average yearly ratios between the frequencies of higher frequency inflections and the frequencies of their original counterparts.

Re-conducting our initial analysis by using inflections that display a higher frequency than the originally selected religious terms confirms previous findings. In particular, we find a significant negative correlation between years and frequencies of religious terms for American English (r = -0.89, p<0.001), British English (r = -0.96, p<0.001), German (r = -0.49, p<0.001), and Italian (r = -0.65, p<0.001). German terms continue to exhibit a positive trend during WWII (r = 0.79, p<0.01).

Despite the benefits of choosing words with more robust historical trends, one disadvantage of the tag “_INF” is the mechanical adjustment of the words’ endings. The inflected terms’ original meanings can sometimes be significantly distorted. One example relates to the Italian word “predica” (sermon), which is far from being represented by the higher frequency modifications “pred**etto**” (aforementioned) and “predica**to**” (predicate). Thus, a manual reconsideration of the words’ meanings, particularly for non-native speakers, is inevitable.

### Procedure IV: Synonyms

To verify that investigated words reflect true underlying concepts rather than idiosyncrasies, re-checking initial findings with several synonyms is a strong robustness check. Pettit [3], for example, argues that especially 1-grams may be risky to analyze due to potential changes in the words’ meanings over time. One example that illustrates Pettit’s [3] concerns is the word “nice”. Nowadays, the word is used to express that something is pleasant. However, in the fourteenth century the term was primarily related to negative quality such as “foolish” or “silly” (see, e.g., https://blog.oxforddictionaries.com/2012/10/01/change-in-word-meanings/). Using synonyms reduces the impact of a particular word and therefore decreases the probability of incorrect assumptions due to, e.g., varying meanings, idiosyncrasies, messy metadata, and OCR errors. Younes and Reips [14] introduced the use of synonyms for the German corpus by recommending the collection of the first three one-word synonyms out of the semantically most adequate grouping (i.e., only synonyms of “date” as a type of fruit, not as “going out”), listed in the *Duden Synonymwörterbuch*, the standard reference for the German language. In the current study, we followed this procedure for selecting German synonyms. In addition, we collected the first three one-word English synonyms for the most adequate grouping using the standard reference for English that is *Roget’s Thesaurus*. For selecting Italian synonyms, we used and recommend *Zanichelli’s Sinonimi e Contrari*.

Table C in S1 Appendix presents an overview of collected synonyms. We obtained at least one synonym in 95% of all cases. A dictionary-based approach may however not always be the optimal choice to obtain synonyms. Although it avoids an arbitrary selection of words, not for every word there are synonyms and some of the obtained synonyms are often used in a non-related context. Hence, dependent on the investigated language and particularly the topic, it might be helpful to consult several native speakers to suggest (additional) synonyms or rate the obtained synonyms according to their suitability. In accordance with this notion, we let our synonyms rate by two native speakers per language on a scale of 0 (no synonym) to 10 (perfect synonym). The average rating for all synonyms over all languages was relatively high, m = 7.5 (6.8 for English, 7.1 for German, and 8.5 for Italian).

Google Ngram provides frequencies for all synonyms except for the German word “Brandopferstätte” (altar). Analyzing correlations for our set of synonyms, we can confirm the results obtained for the sample of original words. In particular, we find significant negative correlations between years and American English (r = -0.96, p<0.001), British English (r = -0.93, p<0.001), German (r = -0.92, p<0.001), and Italian (r = -0.81, p<0.001) word frequencies. With respect to WWII, German terms continue to exhibit a positive trend, which is however not significant (r = 0.12, p>0.1).

### Procedure V: Standardization of word frequencies

In spite of Michel et al.’s [4] argument that “the most robust historical trends are associated with frequent n-grams” ([4], Supplementary Online Material, p.12), high frequency words significantly drive average results if their frequency is relatively larger than the frequencies of other words that are summed up. In order to account for the influence of individual words, several studies transform word frequencies into z-scores prior to summing them (see, e.g., [6,9,15]). Although this procedure mitigates the disproportionately large influence of single words by giving each word an equal weight, it does not account for the varying size of the corpora, i.e., the influx of data over time. To address this concern, Acerbi et al. [20] and Bentley et al. [41] suggest normalizing word frequencies by expressing a word’s frequency relative to the frequency of a very common word.

In this section, we demonstrate that the combination of z-scoring and normalizing raw word frequencies by common words is a beneficial procedure that can mitigate biased estimations. By themselves, z-scoring and normalizing by a common word either account for unequal weights or data-related trends, respectively. To address both concerns simultaneously, we propose a new procedure that accounts for data-related trends and gives each word an equal weight. In particular, we recommend subtracting the summed z-scored frequencies of various very common words obtained from Lin et al. [5], from the summed z-scored frequencies of the original terms. This procedure is beneficial for several reasons. First, we can mitigate any disproportionately large influence of single words by giving each of the original terms an equal weight. Second, we do not only focus on one common word, whose pattern might deviate from the pattern of other very common words, but on various common words. Third, by also z-scoring the set of common words, we further ensure to treat all common words equally.

In the following, we highlight the differences in results between several standardization procedures. In particular, we compare correlation coefficients between time and religious word frequencies considering (I) summed raw frequencies, (II) summed z-scores of raw frequencies, (III) summed raw frequencies that were previously normalized by the raw frequency of a very common word, and (IV) summed raw frequencies that were previously normalized by the summed raw frequencies of various very common words. In a further step, we present the results for (V) summed z-scores of raw frequencies that were previously normalized by the raw frequency of a very common word and (VI) summed z-scores of raw frequencies which were previously normalized by the summed raw frequencies of various very common words. Finally, we present the correlation coefficients for summed z-scores of raw frequencies that were normalized by subtracting the summed z-scores of raw frequencies of various very common words (VII).

We calculate z-scores as

$$z_{t}=\frac{w_{t}-\mu }{\sigma }.$$ (1)

*w*_*t*_ represents the frequency of a religious word *w* in year *t*, *μ* the mean of *w*_*t*_, and *σ* the standard deviation of *w*_*t*_.

To account for the influx of data, we first identified the most common word in each corpus by manually comparing the frequencies for each of the most common words as indicated by Lin et al. [5]. Lin et al.’s [5] list contains the 16 most common words in each language, equally split into the word classes *adjectives*, *prepositions*, *adverbs*, *conjunctions*, *articles*, *nouns*, *pronouns*, and *verbs*. The most common word in the American and British English corpora is “the”, in the German corpus it is “der”, and in the Italian corpus it is “di”.

**Fig 3:**
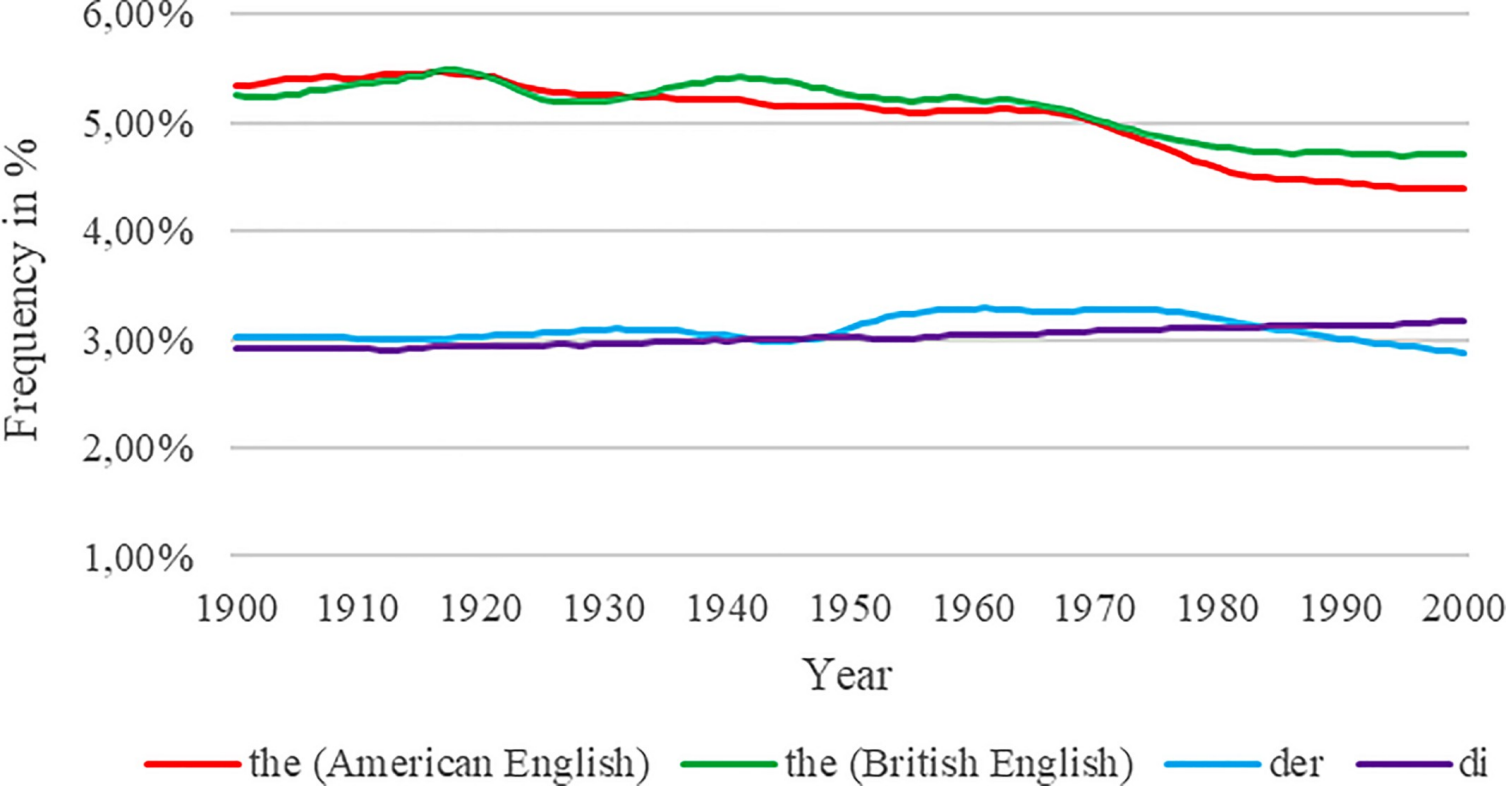
Frequencies for most frequent words. Frequencies for the most frequent words in the American English, British English, German, and Italian Google Ngram corpora.

Fig 3 shows the extent of variation across time for the most frequent word in each corpus. Theoretically, the relative frequency of these neutral words should remain rather constant over time. However, even these words are subject to a trend. For example, the word “the” decreased by approximately 1% (which is ca. 20% of the overall frequency) over the last century, whereas the word “di” exhibited a 0.3% increase (which is ca. 10% of the overall frequency) over the same time. Hence, Fig 3 reinforces the concern that the influx of data should be taken into account by applying an appropriate standardization procedure to avoid biased estimations.

To obtain a reference set of various common words, we consider Lin et al.’s [5] total list of common words and compare it to Hughes et al.’s [42] list of neutral words, i.e., words with little or no specific meaning. This procedure ensures that results are not driven by any specific underlying context of the common words. In particular, we select all words that appear on both lists but refrain from including pronouns, based on their categorization as content-full words by previous literature (see, e.g., [7,11,18,37,38]). Because Hughes et al.’s [42] list comprises only English terms, we translated Lin et al.’s [5] most common German and Italian words to English using PONS. We then searched for the translated words on Hughes et al.’s [42] word list. Applying this procedure, we obtain the words “other”, “such”, “of”, “in”, “not”, “when”, “and”, “or”, “the”, “a”, “is”, and “was” for American and British English. For German, we identified “anderen”, “ersten”, “in”, “von”, “auch”, “so”, “und”, “daß”, “der”, “die”, “ist”, and “werden”. Finally, our list of common Italian words comprises “stesso”, “di”, “in”, “non”, “piú”, “che”, “ed”, “la”, “il”, and “é” (see Figures O-R in S1 Appendix for visual inspection). Table 3 presents the correlation coefficients for the different standardization procedures and highlights how sensitive results can be, depending on an author’s choice of methodology.

**Table 3:** Correlation coefficients for different standardization procedures.

	(I)	(II)	(III)	(IV)	(V)	(VI)	(VII)
Original Words	Σ Raw	Σ z-scores	$\sum \frac{Raw}{Most\;common}$	$\sum \frac{Raw}{\Sigma \;Most\;common}$	Σ z−scores of $\left[\frac{Raw}{Most\;common}\right]$	Σ z−scores of $\left[\frac{Raw}{\Sigma \;Most\;common}\right]$	Σ z−scores − Σ z−scores of most common
American English	r = -0.89, p<0.001	r = -0.88, p<0.001	r = -0.71, p<0.001	r = -0.77, p<0.001	r = -0.70, p<0.001	r = -0.76, p<0.001	r = -0.49, p<0.001
British English	r = -0.96, p<0.001	r = -0.94, p<0.001	r = -0.95, p<0.001	r = -0.96, p<0.001	r = -0.91, p<0.001	r = -0.92, p<0.001	r = -0.81, p<0.001
German WWII	r = -0.47, p<0.001 r = 0.79, p<0.01	r = -0.29, p<0.01 r = 0.87, p<0.001	r = -0.51, p<0.001 r = 0.81, p<0.001	r = -0.46, p<0.001 r = 0.79, p<0.01	r = -0.32, p<0.001 r = 0.89, p<0.001	r = -0.26, p<0.01 r = 0.87, p<0.001	r = -0.13, p>0.1 r = 0.85, p<0.001
Italian	r = -0.64, p<0.001	r = 0.24, p<0.05	r = -0.76, p<0.05	r = -0.58, p<0.001	r = -0.01, p>0.1	r = 0.35, p<0.001	r = 0.51, p<0.001

Whereas results for the American and British English corpora remain relatively stable, Model VII shows that the significance for the German corpus vanishes by accounting simultaneously for unequal weights and data-related time trends. For the Italian corpus results are very inconsistent. As indicated by Model I, considering raw frequencies, we find a significant negative trend over time. If we z-score raw frequencies (Model II), we obtain a positive trend that, however, turns negative if we normalize frequencies beforehand by a very common word (Model III). Finally, by addressing both, unequal weights and the influx of data (Model VII) Italian terms exhibit a positive trend over time. As the main religion in the US, UK, Germany, and Italy has been Christianity, we added a further set of 14 Christian-specific nouns from Ritter and Preston [35] in an additional robustness check (i.e., the words baptism, Bible, Christmas, church, commandments, communion, cross, gospel, Jesus, messiah, preacher, Sabbath, salvation, and worship). Replicating the results in Table 3, Model VII, for a total set of 34 religious terms per language, we obtain similar results as for the original set of religious words for American English (r = -0.51, p<0.001), British English (r = -0.84, p<0.001), German (r = -0.20, p<0.05), WWII (r = 0.80, p<0.01), and Italian (r = 0.56, p<0.001).

### Composite analysis

In this section, we combine our suggested procedures by using higher frequency words, synonyms, and the standardization procedure that accounts for unequal weights and the influx of data. In particular, we re-calculated previous analyses including the sets of additional synonyms and using the tag _INF to identify the most frequent words for our sets of original terms and synonyms (see Table D in S1 Appendix for an overview of corresponding higher frequency synonyms). Table 4 presents correlation coefficients for different standardization procedures using higher frequency words for original terms and additional synonyms.

**Table 4:** Correlation coefficients for different standardization procedures using higher frequency words and synonyms.

	(I)	(II)	(III)	(IV)	(V)	(VI)	(VII)
Original Terms + Synonyms	Σ Raw	Σ z-scores	$\sum \frac{Raw}{Most\;common}$	$\sum \frac{Raw}{\Sigma \;Most\;common}$	Σ z−scores of $\left[\frac{Raw}{Most\;common}\right]$	Σ z−scores of $\left[\frac{Raw}{\Sigma \;Most\;common}\right]$	Σ z−scores − Σ z−scores of most common
American English	r = -0.92, p<0.001	r = -0.83, p<0.001	r = -0.77, p<0.001	r = -0.83, p<0.001	r = -0.60, p<0.001	r = -0.68, p<0.001	r = -0.72, p<0.001
British English	r = -0.95, p<0.001	r = -0.91, p<0.001	r = -0.94, p<0.001	r = -0.95, p<0.001	r = -0.87, p<0.001	r = -0.89, p<0.001	r = -0.89, p<0.001
German WWII	r = -0.73, p<0.001 r = 0.66, p<0.05	r = -0.33, p<0.001 r = 0.86, p<0.001	r = -0.76, p<0.001 r = 0.72, p<0.01	r = -0.74, p<0.001 r = 0.65, p<0.05	r = -0.36, p<0.001 r = 0.88, p<0.001	r = -0.29, p<0.01 r = 0.86, p<0.001	r = -0.26, p<0.01 r = 0.85, p<0.001
Italian	r = -0.76, p<0.001	r = -0.28, p<0.01	r = -0.84, p<0.001	r = -0.72, p<0.001	r = -0.49, p<0.001	r = -0.19 p<0.1	r = -0.14, p>0.1

The results reinforce the significant negative trend for German terms over time suggesting that prior results were affected by the set of original words. Indeed, re-running Model VII by only considering synonyms also displays a significant negative trend (r = -0.20, p<0.05). Thus, particularly the consideration of additional synonyms provides a more robust picture. Further, for Italian terms, a positive trend (as suggested by using the standardization procedure that accounts for an influx of data and unequal weights) cannot be confirmed. Instead, conducting the composite analysis, we do not find any significant trend (Model VII). In fact, we even obtain a significant negative trend over time by re-running Model VII but only considering synonyms (r = -0.36, p<0.001). As a final robustness check, we re-run Model VII, by dropping synonyms that received an average rating of less than the mean rating of 7.5. Results do not change for American English (r = -0.64, p<0.001), British English (r = -0.89, p<0.001), German (r = -0.23, p<0.05), WWII (r = 0.89, p<0.001), and Italian (r = -0.09, p>0.1).

Overall, this analysis strongly emphasizes that only the combination of different methodological procedures mitigates biased estimations and therefore prevents researchers from deriving wrong and undifferentiated assumptions.

### Censorship and propaganda

Finally, we discuss the importance of taking any potential censorship and propaganda into account. Michel et al. [4] address these issues by stressing, for example, the decreasing popularity of the Jewish artist Marc Chagall in the German corpus during the Nazi regime, the absence of the 1976 and 1989 Tiananmen Square incidents in the Chinese corpus, and the reflection of the Hollywood Ten’s blacklisting in the American corpus. In this section, we aim to increase awareness that censorship and propaganda may not only affect certain people or historical events but whole subject-areas. In particular, we graphically illustrate the impact of the Soviet regime’s (1922–1991) enforcement of secularization and religious persecution on the Russian corpus by plotting the overall frequencies for the original religious terms translated to Russian. For this reason, we translated our original English terms to Russian using PONS. Two native speakers re-checked and then confirmed the translations.

**Fig 4:**
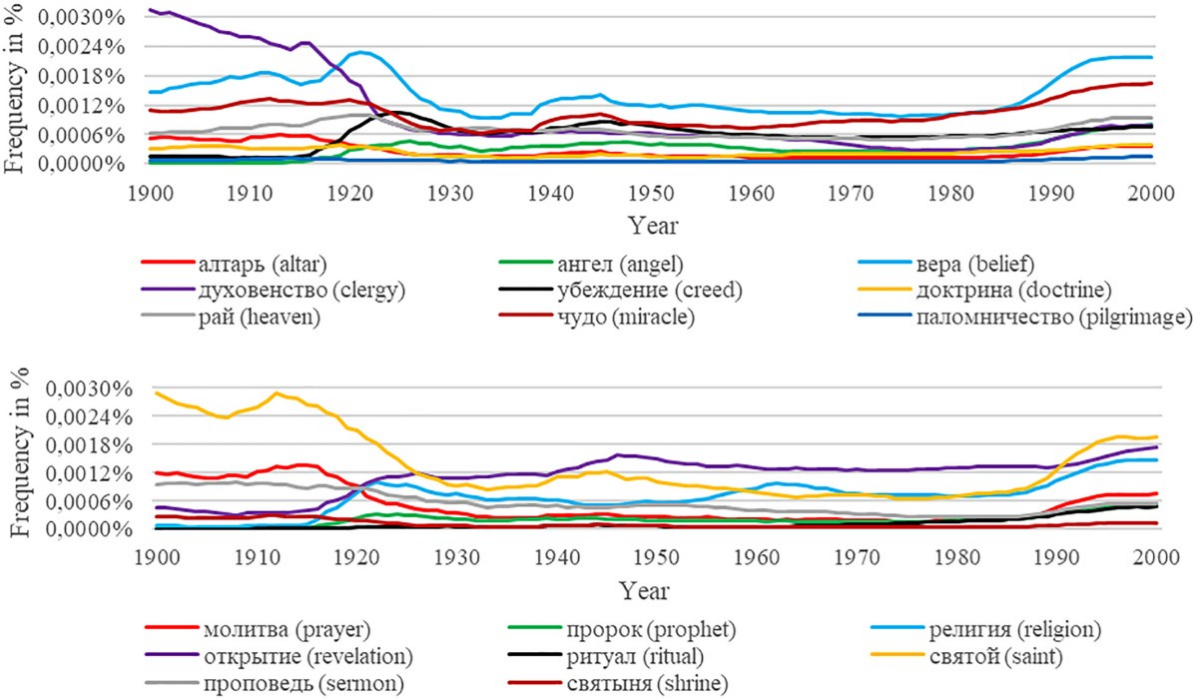
Frequencies of religious terms translated to Russian.

Fig 4 shows the results. The graphs include all of the original translated terms except for “Бог” (God), “душа” (soul), and “дух” (spirit). We did not include these words due to scaling differences. However, they display a similar curvature.

The graphs highlight a drop around the 1920s, followed by rather flat movement until the 1990s, where the frequencies start to rise again. In this respect, they likely mirror the Soviet regime’s objective to eliminate religion. Because there is evidence that Russians acted out religion secretly rather than becoming atheists (see, e.g., [31,43]), there is a gap between what was prevalent in people’s minds and what was artificially created. Therefore, the assumptions which we would derive from visual inspection and also by calculating a correlation coefficient, would mirror the development of the importance of religion for the Soviet Union’s population only undifferentiated and incomplete. Hence, a country’s historical context must always be taken into account. In case of any potential concerns about distorted time periods, we recommend following Hamamura and Xu [11] and investigate sub-periods (e.g., decades) to iteratively discover cultural trends.

## Discussion

Google Ngram allows for hands-on quantification of cultural change using millions of books. In spite of the tool’s unique opportunities for research purposes, several studies justifiably emphasize the existence of potential problems. With this paper, we contribute to the ongoing debate of weighting the tool’s advantages and disadvantages against each other. To the best of our knowledge, this is the first summary of the tool’s limitations that comes with a set of methodological procedures that are suited to improve the reliability of results. In this guideline we present five methodological procedures, a roadmap on how to use them in combination, and a way to check for influences of censorship and propaganda. In particular, to test the universality of a certain theory or hypothesis and to examine derived results cross-culturally, we propose (I) the use of different language corpora. Further, (II) using the fiction corpus, which is not heavily impacted by scientific literature, allows researchers to reinforce results obtained for the American and British English corpora. We additionally recommend to (III) compare frequencies of words and their inflections using the feature “_INF”. We further advise researchers to (IV) make use of synonyms to diminish the probability of wrong assumptions caused by the prevalence of, e.g., OCR errors, flaws related to messy metadata, idiosyncrasies, or the disproportionately large influence of single authors’ contributions. Finally, we also strongly recommend to (V) apply a standardization procedure that accounts for both, the influx of data and unequal weights.

In addition to the individual benefits of each procedure, we strongly advise researchers to use them in combination. As shown by a composite analysis, the most robust results are obtained by combining several procedures. So far, most prior studies did not challenge their results by taking different procedures into account. Based on the literature review displayed in Table A in S1 Appendix, only approximately 3% of previous studies considered both, the use of synonyms and any form of standardization procedure. None of the studies took the particular use of higher frequency inflections into account. As with any new and evolving methodology, many such procedures are only discovered as beneficial over time. Our guideline demonstrates how sensitive results can be to specific choices an author makes and how to resolve such dependencies. For example, by focusing on raw frequencies or by applying a standardization procedure that only accounts for the influx of data over time, we obtain a significant negative trend for Italian religious terms (see Table 3, Model I and III). However, accounting for unequal weights (see Table 3, Model II) leads to the impression that in Italy, religion became more important over time. Furthermore, taking unequal weights and time trends simultaneously into account, the significant positive trend remains only for our set of original terms (see Table 3, Model VII) but turns negative for our set of synonyms. Finally, combining words of comparatively high frequency and the most advanced standardization procedure with an additional set of synonyms, no significant trend is observable any longer (see Table 4, Model VII). Consequently, this example illustrates that only analyzing Google Ngram data in pre-determined appropriate ways can prevent authors from deriving “first-glance” assumptions that may turn out as not valid when a more robust analysis procedure is applied.

Finally, by the example of forced secularization during the Soviet regime, we draw attention to the danger of biased estimations that can arise from censorship and propaganda. However, we would like to point out that what we believe is censorship might be also attributable to the lack of metadata. In particular, Koplenig [44] shows that with the lack of proper metadata, it cannot be ruled out that trends arise due to changes in the composition of the underlying data. Thus, some of the trends we observe for the Russian corpus may not necessarily result from censorship as part of forced secularization, but some change in the data.

By applying all of the above-mentioned procedures to religious 1-grams, this study further contributes to the body of research that investigates the development of collectivism and individualism (see, e.g., [30] for a review) as well as to the literature that discusses religion as a coping strategy during crises (see, e.g., [34] for a review). In particular, we study the cross-cultural development of religious trends for the years 1900 to 2000, with a particular focus on the development in times of crisis such as WWII. Except for the Italian corpus, our analyses reveal a relative overall decrease for religious terms. With respect to WWII, German terms display an upward trend, indicating the importance of religion during a severe crisis along with the Germans’ movement towards a more collectivistic society during the war [14]. The trend towards an increased expression of religion during a severe time of crisis is, however, not only observable in the German corpus. Additional analyses further reveal a significant positive and robust trend for Italian religious terms. The positive trend for Italian terms likely relates to the alliance between Nazi-Germany and Italy during WWII. In contrast, for the American and British English corpora we do not find such a reversal but a constant and significant negative time trend over the whole observation period. Although both countries also participated in the war, we believe that in contrast to Italy, results strongly relate to the larger distance of the respective countries’ population to the main war zone (see Table E in S1 Appendix for an overview of detailed results).

Our study is not without limitations. First, in spite of our suggestions on how to mitigate the influence of the potentially large number of scientific texts in the Google Ngram’s corpora, we did not discuss concerns related to the corpora’s general level of diversity. Although the developers have addressed this issue by providing a corpus with a better-balanced text collection, i.e., “English One Million”, we do not recommend the use of this corpus because it has never been updated and is therefore considered to be per se more error prone. Nevertheless, re-calculating previous analyses by using the corpus “English One Million”, we obtain similar results. Second, we used the new updated corpora to exploit the advantages of improved OCR and better underlying library and publisher metadata. However, as suggested by Twenge et al. [9], the new corpora might entail errors that the old corpora did not capture. We therefore re-examined all analyses using the old corpora. Overall, previous findings are confirmed. However, there was no Italian corpus before the update. Finally, we agree that the key assumption of Google Ngram studies, i.e., that print culture represents culture as a whole, is certainly an undifferentiated view that may not always hold.

Overall, Google Ngram has allowed scholars to shed further light on various topics such as gender differences [17,18,19], emotions [20,21,22,45], personality [23,24], cognition [25,26,27], hypnosis and psychotherapy [46], moral values [47], education [48], nature [49], astrology and phrenology [50], and the development of individualism and collectivism (e.g., [6,7,10,51,52]). In this respect, despite limitations, we believe that Google Ngram is a beneficial tool for research purposes and that the procedures presented in this guideline can reinforce the reliability of derived results.

## Supporting information

S1 Appendix (PDF)

## References

[1] Gooding P. (2012). Mass digitization and the garbage dump: The conflicting needs of quantitative and qualitative methods. *Literary and Linguistic Computing*, 28(3), 425–431.

[2] Pechenick E. A., Danforth C. M., & Dodds P. S. (2015). Characterizing the Google Books corpus: Strong limits to inferences of socio-cultural and linguistic evolution. *PloS One*, 10(10), e0137041 10.1371/journal.pone.0137041 26445406 PMC4596490

[3] Pettit M. (2016). Historical time in the age of big data: Cultural psychology, historical change, and the Google Books Ngram Viewer. *History of Psychology*, 19(2), 141 10.1037/hop0000023 27100927

[4] Michel J. B., Shen Y. K., Aiden A. P., Veres A., Gray M. K., Pickett J. P., et al (2011). Quantitative analysis of culture using millions of digitized books [With Supporting Online Material]. *Science*, 331(6014), 176–182. 10.1126/science.1199644 21163965 PMC3279742

[5] Lin Y., Michel J. B., Aiden E. L., Orwant J., Brockman W., & Petrov S. (2012, 7). Syntactic annotations for the Google Books Ngram corpus In *Proceedings of the ACL 2012 System Demonstrations* (pp. 169–174). Association for Computational Linguistics.

[6] Twenge J. M., Campbell W. K., & Gentile B. (2012a). Increases in individualistic words and phrases in American books, 1960–2008. *PloS One*, 7(7), e40181. 22808113 10.1371/journal.pone.0040181 PMC3393731

[7] Twenge J. M., Campbell W. K., & Gentile B. (2013). Changes in pronoun use in American books and the rise of individualism, 1960–2008. *Journal of Cross-Cultural Psychology*, 44(3), 406–415.

[8] Kesebir P., & Kesebir S. (2012). The cultural salience of moral character and virtue declined in twentieth century America. *The Journal of Positive Psychology*, 7(6), 471–480.

[9] Twenge J. M., VanLandingham H., & Campbell W. K. (2017). The seven words you can never say on television: Increases in the use of swear words in American books, 1950–2008. *SAGE Open*, 7(3), 2158244017723689.

[10] Greenfield P. M. (2013). The changing psychology of culture from 1800 through 2000. *Psychological Science*, 24(9), 1722–1731. 10.1177/0956797613479387 23925305

[11] Hamamura T., & Xu Y. (2015). Changes in Chinese culture as examined through changes in personal pronoun usage. *Journal of Cross-Cultural Psychology*, 46(7), 930–941.

[12] Xu Y., & Hamamura T. (2014). Folk beliefs of cultural changes in China. *Frontiers in Psychology*, 5, 1066 10.3389/fpsyg.2014.01066 25309491 PMC4173642

[13] Zeng R., & Greenfield P. M. (2015). Cultural evolution over the last 40 years in China: Using the Google Ngram Viewer to study implications of social and political change for cultural values. *International Journal of Psychology*, 50(1), 47–55. 10.1002/ijop.12125 25611928

[14] Younes N., & Reips U. -D. (2018). The changing psychology of culture in German‐speaking countries: A Google Ngram study. *International Journal of Psychology*, 53, 53–62. 10.1002/ijop.12428 28474338

[15] Skrebyte A., Garnett P., & Kendal J. R. (2016). Temporal relationships between individualism–collectivism and the economy in Soviet Russia: A word frequency analysis using the Google Ngram corpus. *Journal of Cross-Cultural Psychology*, 47(9), 1217–1235.

[16] Velichkovsky B. B., Solovyev V. D., Bochkarev V. V., & Ishkineeva F. F. (2017). Transition to market economy promotes individualistic values: Analysing changes in frequencies of Russian words from 1980 to 2008. *International Journal of Psychology*. 10.1002/ijop.12411 28075007

[17] Del Giudice M. (2012). The twentieth century reversal of pink-blue gender coding: A scientific urban legend? *Archives of Sexual Behavior*, 41(6), 1321–1323. 10.1007/s10508-012-0002-z 22821170

[18] Twenge J. M., Campbell W. K., & Gentile B. (2012b). Male and female pronoun use in US books reflects women’s status, 1900–2008. *Sex Roles*, 67(9–10), 488–493.

[19] Ye S., Cai S., Chen C., Wan Q., & Qian X. (2018). How have males and females been described over the past two centuries? An analysis of Big-Five personality-related adjectives in the Google English Books. *Journal of Research in Personality*, 76, 6–16.

[20] Acerbi A., Lampos V., Garnett P., & Bentley R. A. (2013). The expression of emotions in 20th century books. *PloS One*, 8(3), e59030 10.1371/journal.pone.0059030 23527080 PMC3604170

[21] Mohammad S. M. (2012). From once upon a time to happily ever after: Tracking emotions in mail and books. *Decision Support Systems*, 53(4), 730–741.

[22] Morin O., & Acerbi A. (2017). Birth of the cool: a two-centuries decline in emotional expression in Anglophone fiction. *Cognition and Emotion*, 31(8), 1663–1675. 10.1080/02699931.2016.1260528 27910735

[23] Roivainen E. (2013). Frequency of the use of English personality adjectives: Implications for personality theory. *Journal of Research in Personality*, 47(4), 417–420.

[24] Roivainen E. (2015). Personality adjectives in twitter tweets and in the Google books corpus. An analysis of the facet structure of the openness factor of personality. *Current Psychology*, 34(4), 621–625.

[25] Ellis D. A., Wiseman R., & Jenkins R. (2015). Mental representations of weekdays. *PloS One*, 10(8), e0134555 10.1371/journal.pone.0134555 26288194 PMC4544878

[26] Hills T. T., & Adelman J. S. (2015). Recent evolution of learnability in American English from 1800 to 2000. *Cognition*, 143, 87–92. 10.1016/j.cognition.2015.06.009 26117487

[27] Virues-Ortega J., & Pear J. J. (2015). A history of “behavior” and “mind”: Use of behavioral and cognitive terms in the 20th century. *The Psychological Record*, 65(1), 23–30.

[28] FAZ. (n.d.). Verteilung der Weltbevölkerung nach Religionen in den Jahren 1900 und 2010. In Statista–Das Statistik Portal. Retrieved on 06.03.2019 from https://de.statista.com/statistik/daten/studie/256878/umfrage/verteilung-der-weltbevoelkerung-nach-religionen/.

[29] Greenfield P. M. (2009). Linking social change and developmental change: shifting pathways of human development. *Developmental Psychology*, 45(2), 401 10.1037/a0014726 19271827

[30] Triandis H. C. (2018). *Individualism and Collectivism*. Routledge.

[31] Inglehart R., & Baker W. E. (2000). Modernization, cultural change, and the persistence of traditional values. *American Sociological Review*, 19–51.

[32] Pargament K. I., Ishler K., Dubow E. F., Stanik P., Rouiller R., Crowe P., et al (1994). Methods of religious coping with the Gulf War: Cross-sectional and longitudinal analyses. *Journal for the Scientific Study of Religion*, 347–361.

[33] Ano G. G., & Vasconcelles E. B. (2005). Religious coping and psychological adjustment to stress: A meta‐analysis. *Journal of Clinical Psychology*, 61(4), 461–480. 10.1002/jclp.20049 15503316

[34] Pargament K. I. (2001). *The psychology of religion and coping*: *Theory*, *research*, *practice*. Guilford Press.

[35] Ritter R. S., & Preston J. L. (2013). Representations of religious words: Insights for religious priming research. *Journal for the Scientific Study of Religion*, 52(3), 494–507.

[36] Oishi S., Graham J., Kesebir S., & Galinha I. C. (2013). Concepts of happiness across time and cultures. *Personality and Social Psychology Bulletin*, 39(5), 559–577. 10.1177/0146167213480042 23599280

[37] Uz I. (2014). Individualism and first person pronoun use in written texts across languages. *Journal of Cross-Cultural Psychology*, 45(10), 1671–1678.

[38] Yu F., Peng T., Peng K., Tang S., Chen C. S., Qian X., et al (2016). Cultural value shifting in pronoun use. *Journal of Cross-Cultural Psychology*, 47(2), 310–316.

[39] Brysbaert M., Keuleers E., & New B. (2011). Assessing the usefulness of Google Books’ word frequencies for psycholinguistic research on word processing. *Frontiers in Psychology*, 2, 27 10.3389/fpsyg.2011.00027 21713191 PMC3111095

[40] Mann, J., Zhang, D., Yang, L., Das, D., & Petrov, S. (2014). Enhanced search with wildcards and morphological inflections in the Google Books Ngram Viewer. In Proceedings of 52nd Annual Meeting of the Association for Computational Linguistics: System Demonstrations (pp. 115–120).

[41] Bentley R. A., Acerbi A., Ormerod P., & Lampos V. (2014). Books average previous decade of economic misery. *PloS One*, 9(1), e83147 10.1371/journal.pone.0083147 24416159 PMC3885402

[42] Hughes J. M., Foti N. J., Krakauer D. C., & Rockmore D. N. (2012). Quantitative patterns of stylistic influence in the evolution of literature. *Proceedings of the National Academy of Sciences*, 109(20), 7682–7686. 10.1073/pnas.1115407109 PMC3356644 22547796

[43] Froese P. (2004). Forced secularization in Soviet Russia: Why an atheistic monopoly failed. *Journal for the Scientific Study of Religion*, 43(1), 35–50.

[44] Koplenig A. (2017). The impact of lacking metadata for the measurement of cultural and linguistic change using the Google Ngram data sets—Reconstructing the composition of the German corpus in times of WWII. *Digital Scholarship in the Humanities*, 32(1), 169–188.

[45] Scheff T. (2015). Toward defining basic emotions. *Qualitative Inquiry*, 21(2), 111–121.

[46] Rossi E., Mortimer J., & Rossi K. (2013). Therapeutic hypnosis, psychotherapy, and the digital humanities: The narratives and culturomics of hypnosis, 1800–2008. *American Journal of Clinical Hypnosis*, 55(4), 343–359. 10.1080/00029157.2012.696078 23724569

[47] Mooijman M., Meindl P., Oyserman D., Monterosso J., Dehghani M., Doris J. M., et al (2018). Resisting temptation for the good of the group: Binding moral values and the moralization of self-control. *Journal of Personality and Social Psychology*, 115(3), 585–599. 10.1037/pspp0000149 28604018

[48] Roivainen E. (2014). Changes in word usage frequency may hamper intergenerational comparisons of vocabulary skills: An Ngram analysis of wordsum, WAIS, and WISC test items. *Journal of Psychoeducational Assessment*, 32(1), 83–87.

[49] Kesebir S., & Kesebir P. (2017). A growing disconnection from nature is evident in cultural products. *Perspectives on Psychological Science*, 12(2), 258–269. 10.1177/1745691616662473 28346112

[50] Genovese J. E. (2015). Interest in astrology and phrenology over two centuries: A Google Ngram study. *Psychological Reports*, 117(3), 940–943. 10.2466/17.PR0.117c27z8 26595286

[51] Li Y., Tan X., Huang Z., & Liu L. (2017). Relationship between collectivism and corruption in American and Chinese books: A historical perspective. *International Journal of Psychology*. 10.1002/ijop.12447 28703329

[52] Grossmann I., & Varnum M. E. (2015). Social structure, infectious diseases, disasters, secularism, and cultural change in America. *Psychological Science*, 26(3), 311–324. 10.1177/0956797614563765 25656275

