# Supplementary material for: Guideline for improving the reliability of Google Ngram studies: Evidence from religious terms
Source: PLoS One. 2019 Mar 22;14(3):e0213554. doi: 10.1371/journal.pone.0213554 (PMC6430395; doi:10.1371/journal.pone.0213554)
Supplement: S1 Appendix — (PDF) [file pone.0213554.s001.pdf]

S1 Appendix

Due to large scaling differences, the Figures A to N of S1 Appendix do not include the words “God”, “Gott”, “Dio”, “Dios”, and “Dieu”, respectively. However, they show a similar curvature.

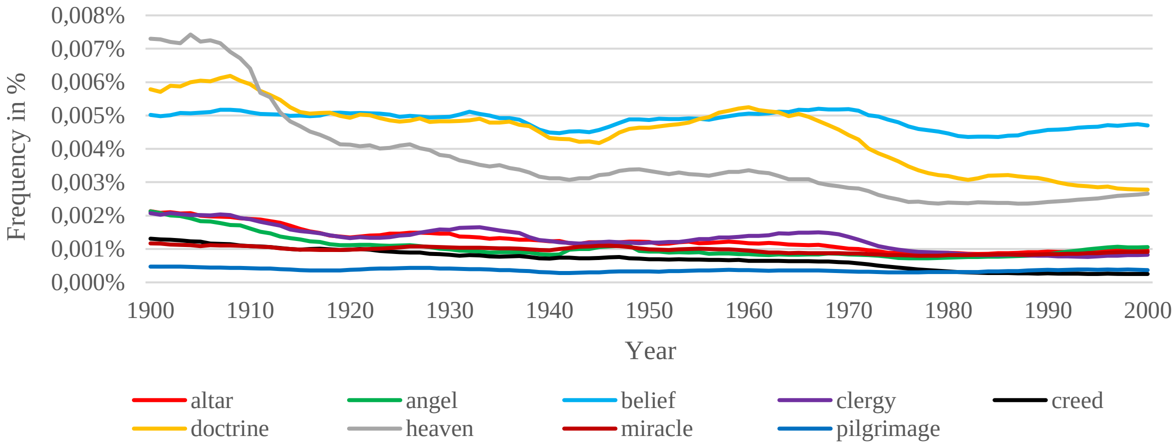

Fig A. Raw frequencies for religious terms using the American English Google Ngram corpus.

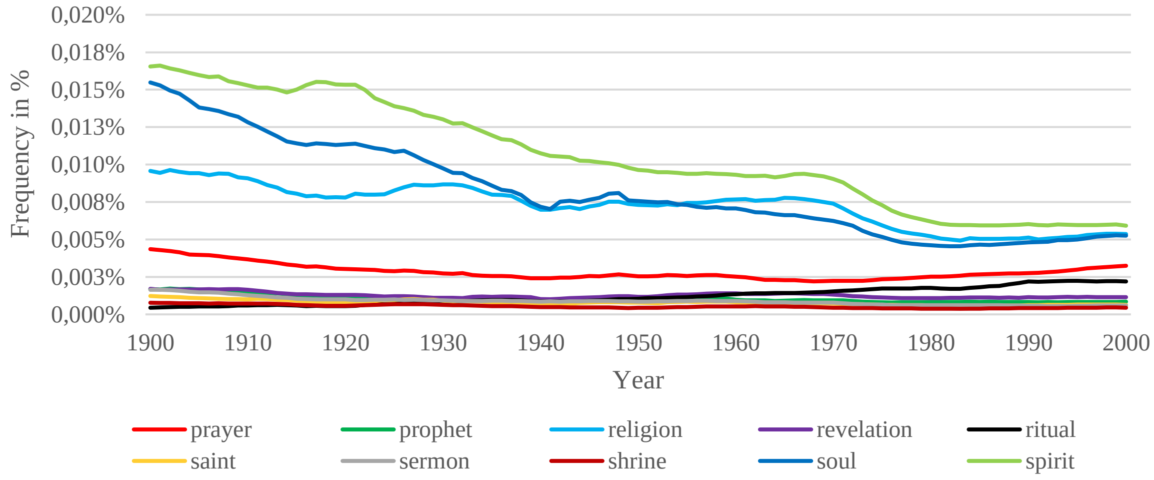

Fig B. Raw frequencies for religious terms using the American English Google Ngram corpus.

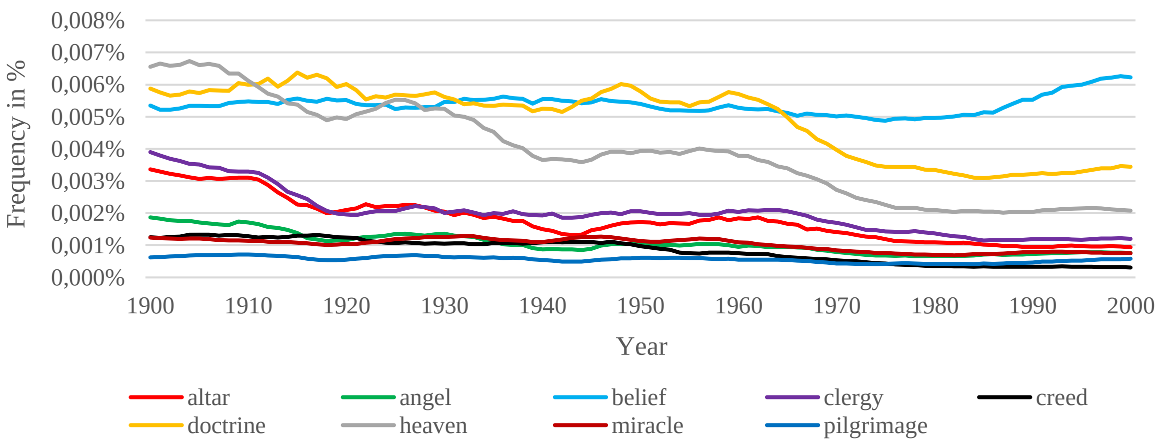

Fig C. Raw frequencies for religious terms using the British English Google Ngram corpus.

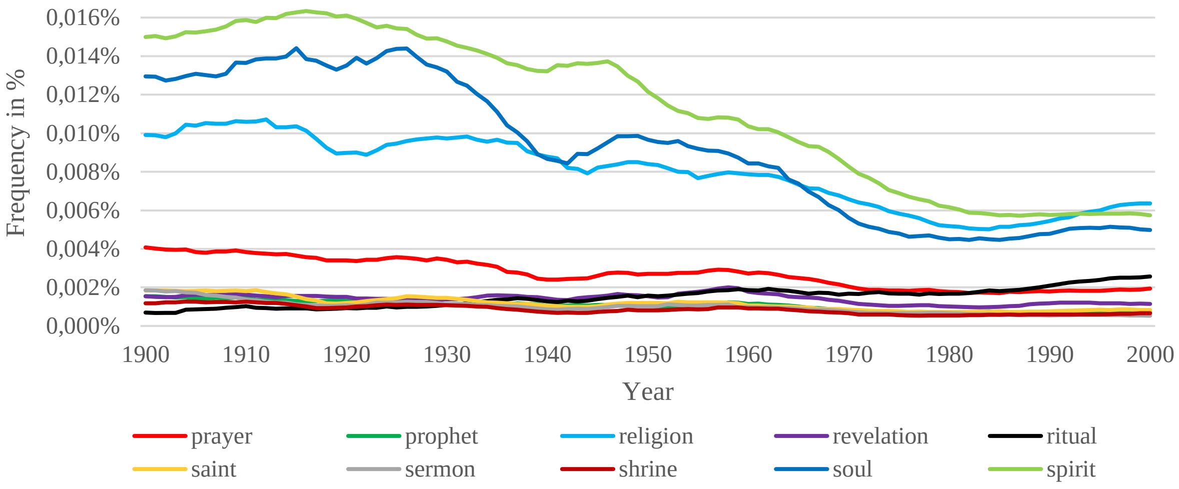

Fig D. Raw frequencies for religious terms using the British English Google Ngram corpus.

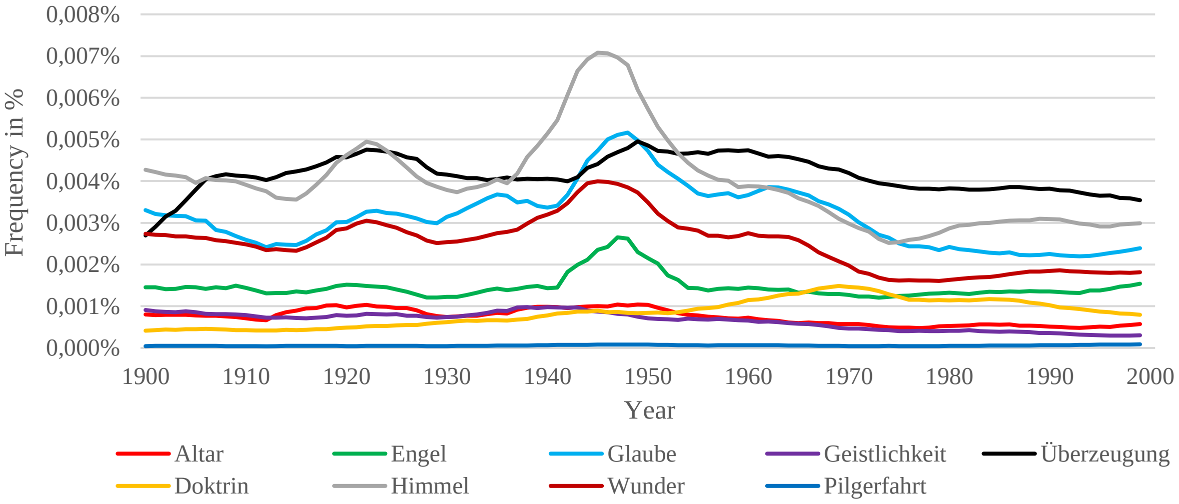

Fig E. Raw frequencies for religious terms using the German Google Ngram corpus.

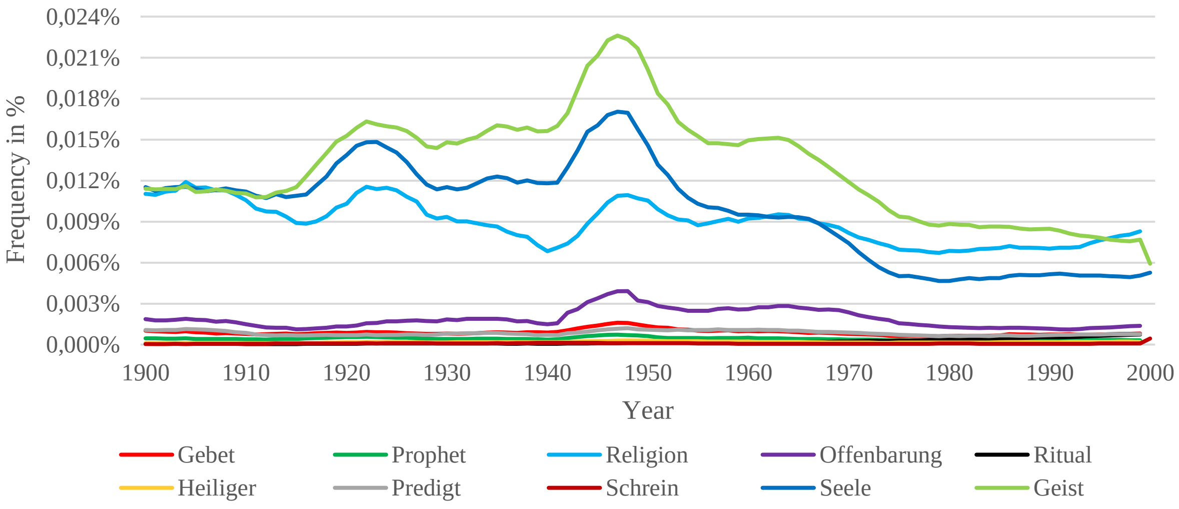

Fig F. Raw frequencies for religious terms using the German Google Ngram corpus.

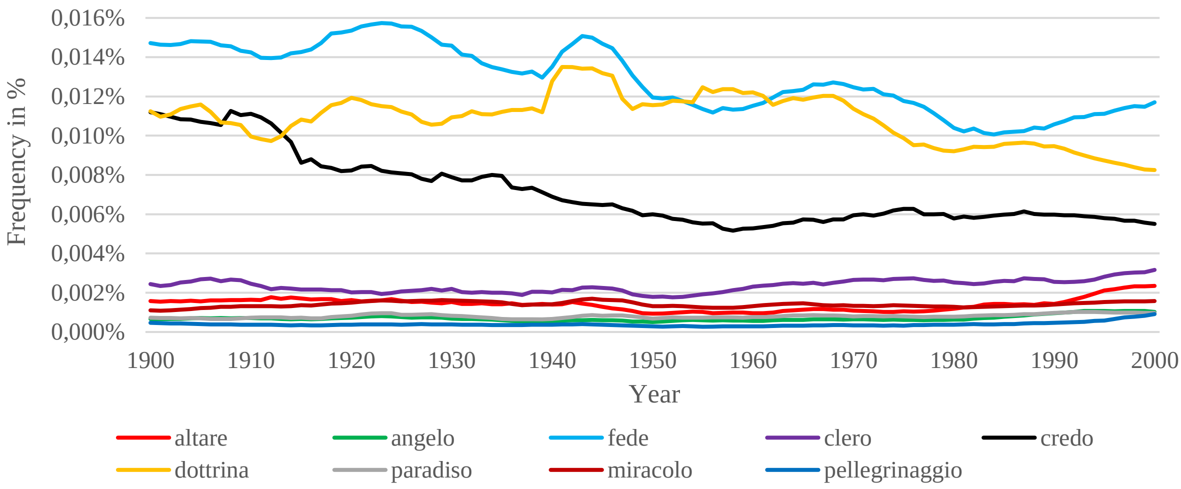

Fig G. Raw frequencies for religious terms using the Italian Google Ngram corpus.

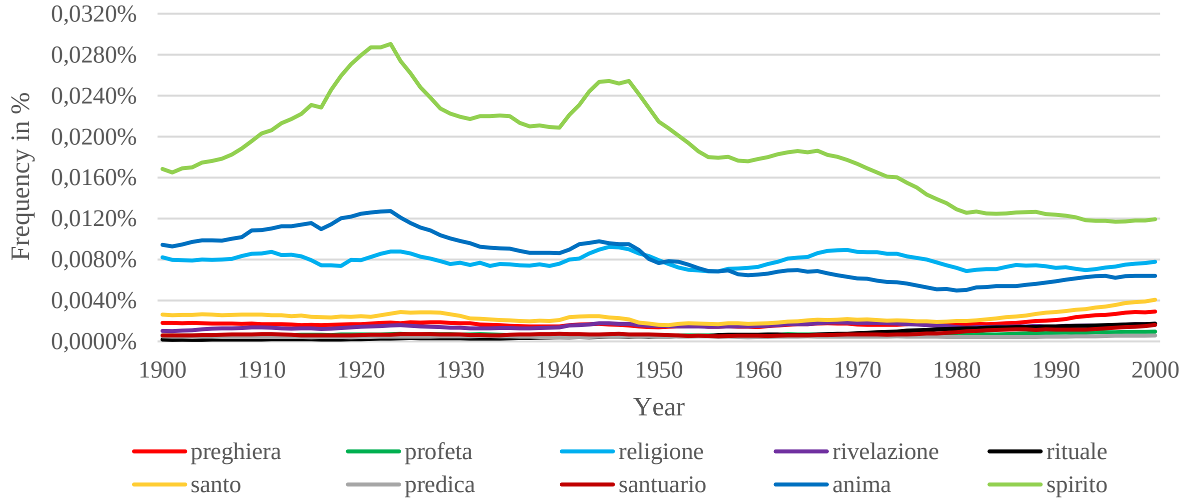

Fig H. Raw frequencies for religious terms using the Italian Google Ngram corpus.

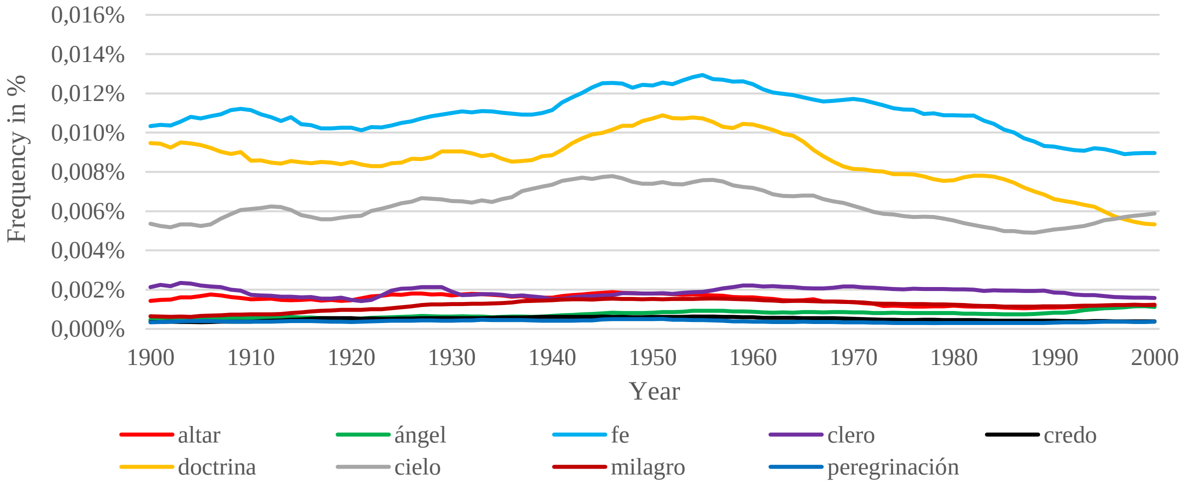

Fig I. Raw frequencies for religious terms using the Spanish Google Ngram corpus.

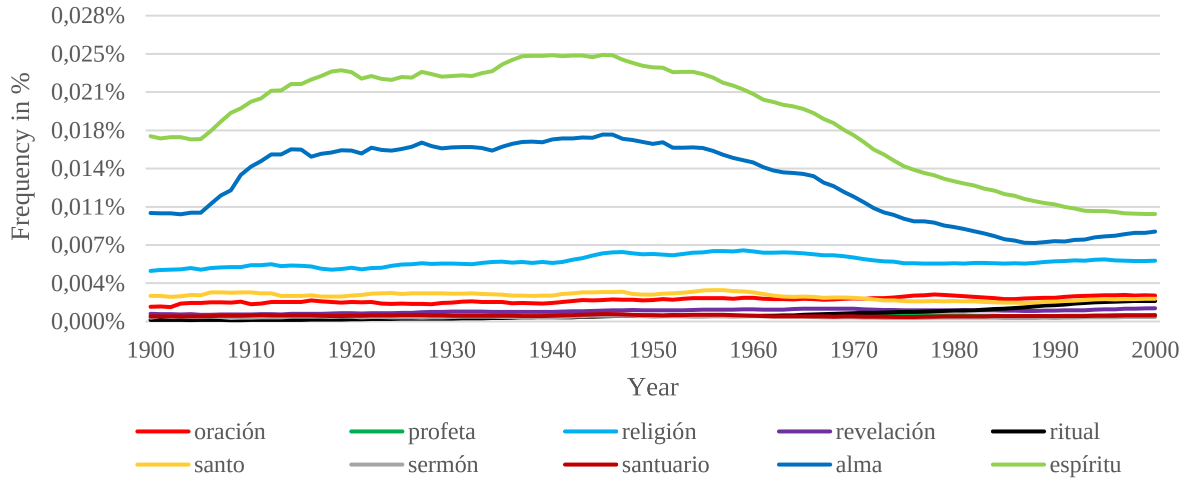

Fig J. Raw frequencies for religious terms using the Spanish Google Ngram corpus.

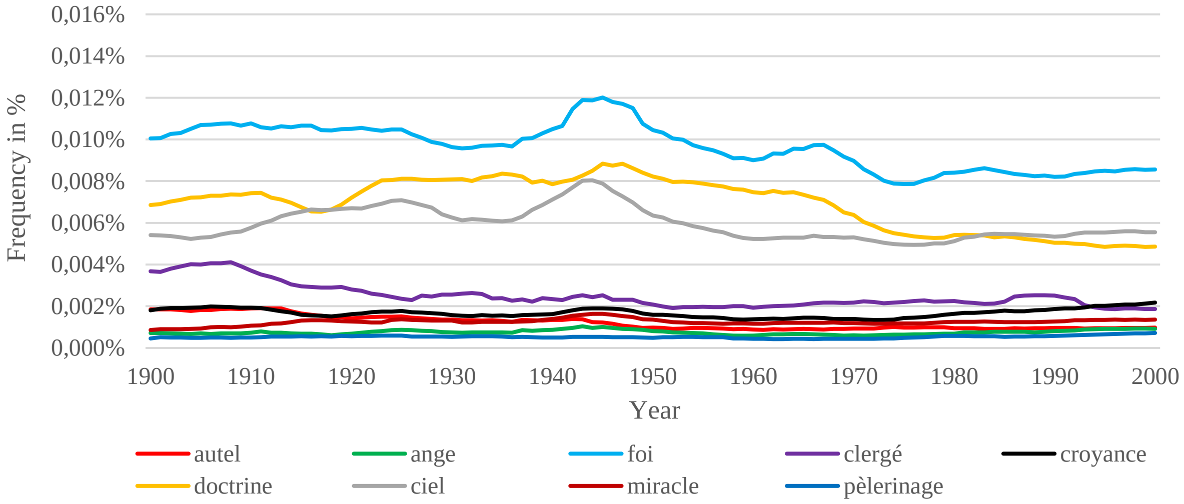

Fig K. Raw frequencies for religious terms using the French Google Ngram corpus.

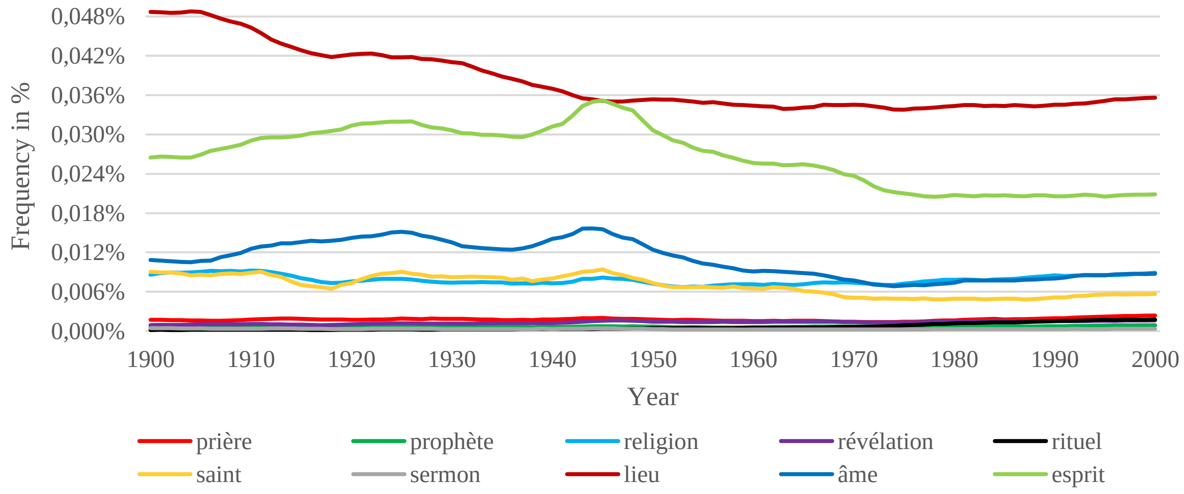

Fig L. Raw frequencies for religious terms using the French Google Ngram corpus.

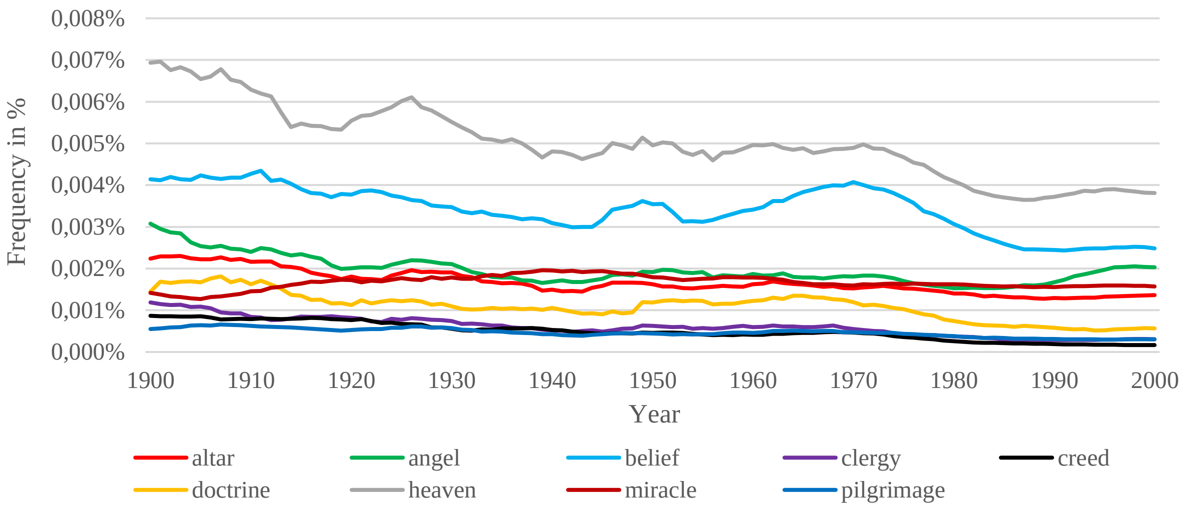

Fig M. Raw frequencies for religious terms using the Fiction English Google Ngram corpus.

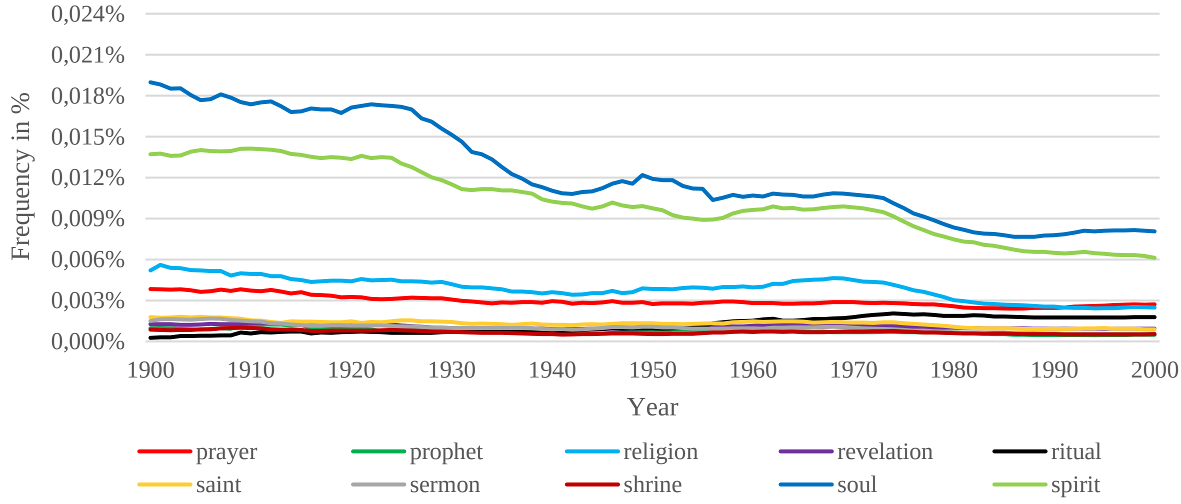

Fig N. Raw frequencies for religious terms using the Fiction English Google Ngram corpus.

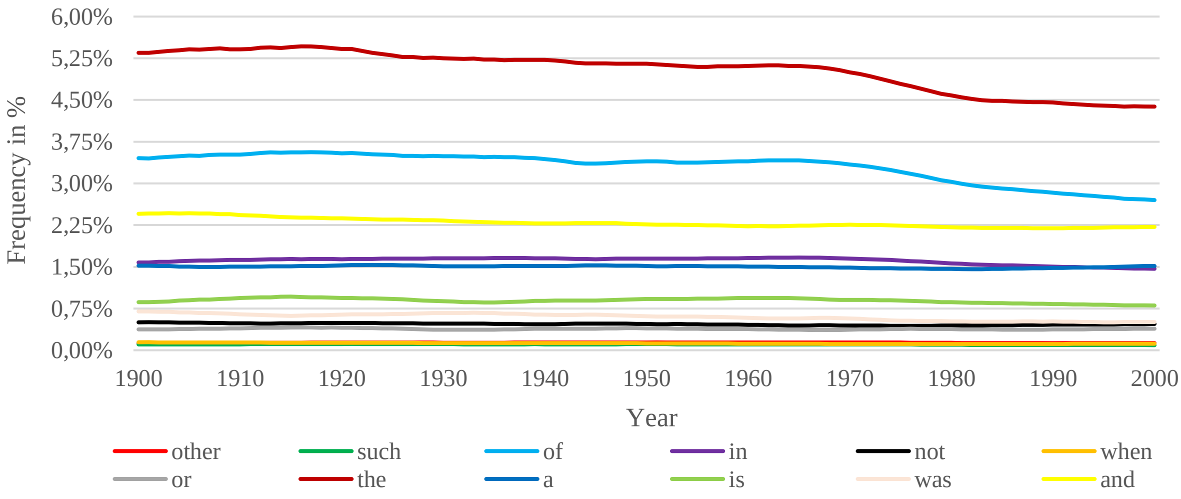

Fig O. Raw frequencies for common words using the American English Google Ngram corpus.

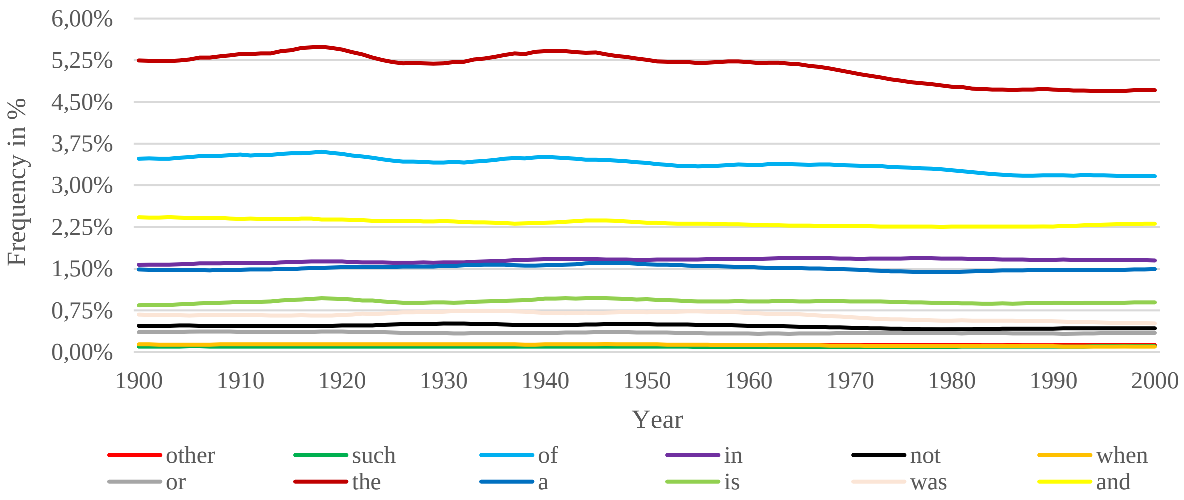

Fig P. Raw frequencies for common words using the British English Google Ngram corpus.

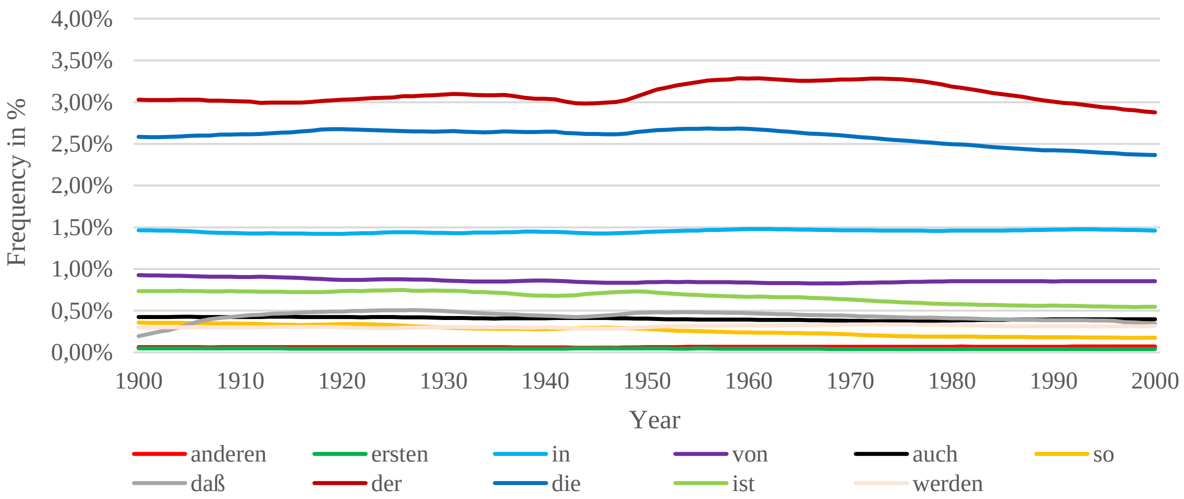

Fig Q. Raw frequencies for common words using the German Google Ngram corpus.

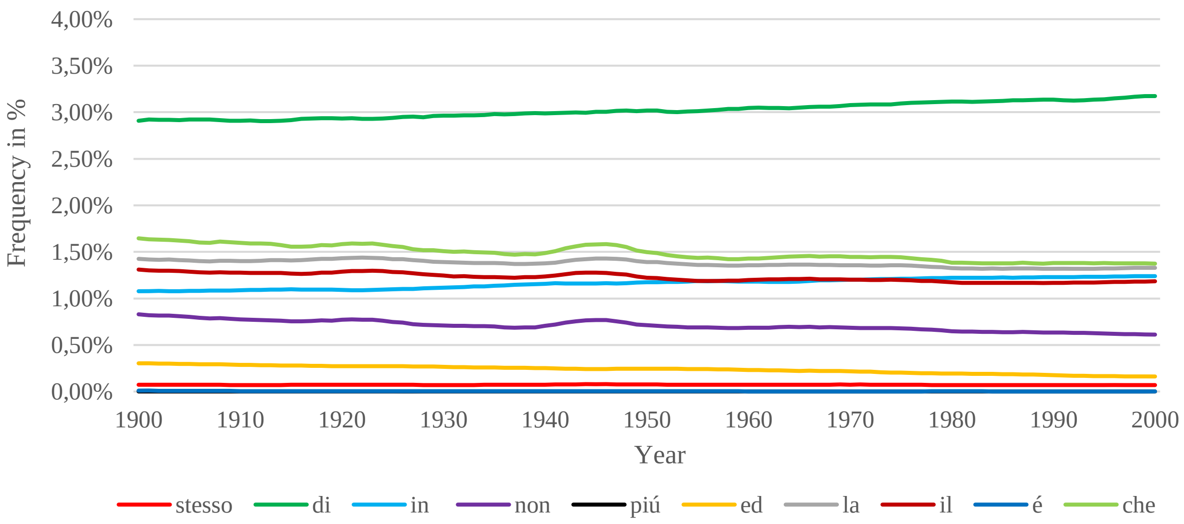

Fig R. Raw frequencies for common words using the Italian Google Ngram corpus.

Table A. Psychological Google Ngram research published between 2010 and 2018.

Journal	Article	Language	Subject
American Journal of Clinical Hypnosis	[46]	English	Other
Archives of Sexual Behavior	[17]	English	Gender
Cognition	[26]	English	Cognition
Cognition and Emotion	[22]	English	Emotions
Current Psychology	[24]	English	Personality
Decision Support Systems	[21]	English	Emotions
Frontiers in Psychology	[12]	Chinese	I/C
International Journal of Psychology	[13]	Chinese	I/C
	[14]	German	I/C
	[16]	Russian	I/C
	[51]	Various	I/C
Journal of Cross-Cultural Psychology	[7]	English	I/C
	[11]	Chinese	I/C
	[15]	Russian	I/C
	[37,38]	Various	I/C
Journal of Personality and Social Psychology	[47]	English	Other
Journal of Positive Psychology	[8]	English	I/C
Journal of Psychoeducational Assessment	[48]	English	Other
Journal of Research in Personality	[19]	English	Gender
	[23]	English	Personality
Personality and Social Psychology Bulletin	[36]	English	Emotions
Perspectives of Psychological Science	[49]	English	Other
PLOS ONE	[6]	English	I/C
	[20]	English	Emotions
	[25]	English	Cognition
Psychological Reports	[50]	English	Other
Psychological Science	[10,52]	English	I/C
Qualitative Inquiry	[45]	Various	Emotions
SAGE Open	[9]	English	I/C
Science	[4]	Various	Other
Sex Roles	[18]	English	Gender
The Psychological Record	[27]	English	Cognition

Note. I/C indicates "Individualism/Collectivism". Literature was collected by conducting a Google Scholar search. The first 50 pages covering the years 2010 to 2018 were taken into account. The search terms were "google ngram books". Only psychological literature was considered.

Table B. Overview of religious terms in Spanish and French.

Original	Spanish	French	Original	Spanish	French
altar	altar	autel	prayer	oración	prière
angel	ángel	ange	prophet	profeta	prophète
belief	fe	foi	religion	religión	religion
clergy	clero	clergé	revelation	revelación	révélation
creed	credo	croyance	ritual	ritual	rituel
doctrine	doctrina	doctrine	saint	santo	saint
God	Dios	Dieu	sermon	sermón	sermon
heaven	cielo	ciel	shrine	santuario	lieu
miracle	milagro	miracle	soul	alma	âme
pilgrimage	peregrinación	pèlerinage	spirit	espíritu	esprit

Table C. Overview of synonyms.

Original	Synonyms English			Synonyms German			Synonyms Italian		
**altar**	sacrarium	sanctuary	prothesis	Opferstätte	Brandopferstätte	X	ara	X	X
**angel**	archangel	seraph	cherub	Cherub	Himmelsbote	Himmelswächter	X	X	X
**belief**	deism	theism	animism	Frömmigkeit	Glaubensüberzeugung	Gläubigkeit	religione	professione	credo
**clergy**	pastor	shepherd	vicar	X	X	X	ecclesiastici	sacerdoti	religiosi
**creed**	credo	dogma	orthodoxy	Anschauung	Ansicht	Betrachtungsweise	dogma	fede	X
**doctrine**	theology	divinity	scholasticism	Dogma	Glaubenssatz	Lehre	principi	teorici	dogma
**God**	Creator	Preserver	Allah	Allwissender	Gottvater	Herr	Iddio	Domineddio	Creatore
**heaven**	paradise	nirvana	zion	Jenseits	Paradies	Himmelreich	eden	cielo	empireo
**miracle**	prodigy	portent	sign	Ausnahmeerscheinung	Geheimnis	Hexenwerk	portento	prodigio	X
**pilgrimage**	rites	mysteries	laud	Wallfahrt	Hadsch	X	viaggio	X	X
**prayer**	orison	devotion	retreat	Bitte	Bittgebet	Dankgebet	orazione	prece	invocazione
**prophet**	priest	archpriest	hierophant	Hellseher	Mahner	Rufer	annunziatore	vate	X
**religion**	creed	dogma	piety	Bekenntnis	Glaube	Glaubenslehre	fede	credo	confessione
**revelation**	divine	apocalypse	disclosure	X	X	X	teofania	X	X
**ritual**	rites	cult	institution	Kult	Ritus	Brauch	cerimoniale	cerimonia	X
**saint**	patron	X	X	Legende	Mythos	Patron	martire	patrono	X
**sermon**	oration	speech	effusion	Kanzelrede	Kanzelwort	Sermon	sermone	omelia	predicazione
**shrine**	temple	fane	pantheon	Heiligenschrein	Reliquiar	Reliquienschrein	tempio	chiesa	sacrario
**soul**	spirit	geist	mind	Empfindungsleben	Gefühlsleben	Gemüt	spirito	X	X
**spirit**	ghost	shades	visitant	Dämon	Erscheinung	Gespenst	anima	X	X

Note. X indicates that no synonym was available. In case singular and plural forms were listed we only considered one form. For the German word "Gott" we did not conisder the pronoun "Er" (he) as a synonym. For the English word "spirit" we did not consider the words "Manes", "lemures", and "zombie" as synonyms.

Table D. Overview of original words and their higher frequency inflections.

American English		British English		German		Italian	
Original	High	Original	High	Original	High	Original	High
orison	orisons	orison	orisons	Cherub	Cherubim	principi	principio
seraph	seraphim	mysteries	mystery	Allwissender	Allwissenden	martire	martiri
mysteries	mystery	shades	shade	Dämon	Dämonen	prece	preci
shades	shade	institution	institutions	Glaubenssatz	Glaubenssätze	omelia	omelie
institution	institutions			Glaube	Glauben	sacerdoti	sacerdote

Note. As noted in the main text, (higher frequency) inflections may change over time.

Table E. Correlation coefficients for WWII using words of highest frequency.

Language	Original Terms	Synonyms	Original Terms + Synonyms
American English	r=-0.88, p<0.001	r=-0.89, p<0.001	r=-0.88, p<0.001
British English	r=-0.95, p<0.001	r=-0.93, p<0.001	r=-0.93, p<0.001
German	r= 0.88, p<0.001	r= 0.81, p<0.001	r= 0.85, p<0.001
Italian	r= 0.41, p>0.1	r= 0.87, p<0.001	r= 0.79, p<0.01

Note. Correlation coefficients are calculated by subtracting the summed z-scores of raw frequencies of various very common words from the summed z-scores of raw frequencies of the original terms and/or synonyms.
